# Supplementary material for: A Home-Treatment Algorithm Based on Anti-inflammatory Drugs to Prevent Hospitalization of Patients With Early COVID-19: A Matched-Cohort Study (COVER 2)
Source: Front Med (Lausanne). 2022 Apr 22;9:785785. doi: 10.3389/fmed.2022.785785 (PMC9073076; doi:10.3389/fmed.2022.785785)
Supplement: Supplementary file 1 [file Data_Sheet_1.pdf]

SUPPLEMENTARY MATERIAL - COVER 2 study

Supplementary Table S1. Covariate balance across overall 'control' and 'recommended treatment' cohorts before and after matching.

	**Control cohort** *(n=3260)*	**Recommended treatment cohort** *(n=108)*	**Standardised Difference before matching*** *(%)*	**Standardised Difference after matching*** *(%)*
**Demographic characteristics**				
Mean age ± SD *(years)*	51.58±14.72	53.1±15.8	10.2	2.6
Males, *n (%)*	1228 (37.67)	46 (42.59)	10.1	17.2
**Comorbidities, *n (%)***				
Cardiovascular disease	159 (4.88)	8 (7.41)	10.6	3.4
Hypertension	591 (18.13)	23 (21.30)	8.0	4.5
Diabetes mellitus	116 (3.56)	1 (0.93)	17.9	18.6
Overweight/Obesity	481 (14.75)	11 (10.18)	13.9	30.9

Data are numbers (percentages).

* Standardised differences between the two groups calculated using 'stddiff' STATA module.

Supplementary Table S2. Demographic and early symptoms associated with COVID-19 illness in the overall ORIGIN control group.

	**Overall** *(n=3260)*
**Demographic characteristics**	
Age, *years*	
18-40	726 (22.27)
41-65	1991 (61.07)
66-75	406 (12.45)
>75	137 (4.20)
Mean age ± SD	51.58±14.72
Males, *n (%)*	1228 (37.67)
**Comorbidities, *n (%)***	
Cardiovascular disease	159 (4.88)
Hypertension	591 (18.13)
Diabetes mellitus	116 (3.56)
Overweight/Obesity	481 (14.75)
**Early symptoms, *n (%)***	
Fever	1932 (59.26)
Myalgia	1501 (46.04)
Tiredness/exhaustion	1890 (57.98)
Dyspnoea	688 (21.10)
Chest pain	476 (14.60)
Headache	1141 (35.00)
Lack of appetite	835 (25.61)
Cough	1195 (36.66)
Sore throat	524 (16.07)
Rhinitis	405 (12.42)
Vomiting/nausea	454 (13.93)
Diarrhoea	672 (20.61)
Red eyes	397 (12.18)
Anosmia	716 (21.96)
Ageusia	1503 (46.10)

Data are numbers (percentages).

Supplementary Table S3. Multivariable logistic regression based on all 3368 patients (3260 included in the 'control' and 108 in the 'recommended treatment' cohort).

	**Odds ratio**	**95% confidence interval**	**P value**
Age *(years)*	0.95	0.94 to 0.97	<0.0001
Male sex	0.26	0.19 to 0.35	<0.0001
Cardiovascular disease	1.60	1.01 to 2.47	0.0408
Hypertension	1.23	0.88 to 1.69	0.2200
Diabetes mellitus	1.51	0.90 to 2.48	0.1083
Overweight/Obesity	2.05	1.45 to 2.88	<0.0001
Treatment (control vs recommended)	8.90	1.92 to 158.37	0.0313

COVER 2 Study Organisation

Members of the COVER 2 Study Organisation includes the following (all in Italy): *Chief Investigator* - Giuseppe Remuzzi (Bergamo); *Study coordinators* - Norberto Perico, Fredy Suter (Bergamo); *Coordinating Centre* – Istituto di Ricerche Farmacologiche Mario Negri IRCCS, Centro di Ricerche Cliniche per le Malattie Rare *Aldo e Cele Daccò*, Ranica (Bergamo); *Study investigators including patients* - Elena Consolaro (Varese), Chiara Moroni (Varese), Umberto Cantarelli (Teramo), Stefania Pedroni (Varese), Maria Vittoria Paganini (Varese), Elena Pastò (Varese), Grazia Pravettoni (Varese), Fredy Suter (Bergamo); *Data collection and processing* – Nadia Rubis, Davide Villa, Olimpia Diadei, Davide Martinetti, Matias Trillini (Bergamo); *Data Analysis* - Annalisa Perna, Tobia Peracchi (Bergamo); *Regulatory Affairs* - Paola Boccardo (Bergamo); *Finalisation of the manuscript*: Norberto Perico, Piero Ruggenenti, Giuseppe Remuzzi (Bergamo).
